# The associations between serum biomarkers and stenosis of the coronary arteries

**DOI:** 10.18632/oncotarget.9645

**Published:** 2016-05-26

**Authors:** Lei Feng, Shiyan Nian, Shu Zhang, Wenbo Xu, Xingfeng Zhang, Dan Ye, Lei Zheng

**Affiliations:** ^1^ Department of Laboratory, People's Hospital of Yuxi City, Yuxi City, Yunnan, P.R. China; ^2^ Department of Laboratory Medicine, Nanfang Hospital, Southern Medical University, Baiyun District, Guangzhou, Guangdong, P.R. China; ^3^ Intensive Care Unit, People's Hospital of Yuxi City, Yuxi City, Yunnan, P.R. China; ^4^ Department of Laboratory, The Sixth Affiliated Hospital of Kunming Medical University, Yuxi City, Yunnan, P.R. China

**Keywords:** coronary heart disease, serum biochemical indices, risk factors, binary multiple logistic regression analysis, ordinal multiple logistic regression analysis, Pathology Section

## Abstract

Serum biochemical indices reflect dynamic physiological and pathophysiological processes within the body, the associations between these markers and the number of stenotic coronary arteries have been rarely studied. 627 healthy controls and 1,049 coronary heart disease (CHD) patients were sequentially recruited in our hospital. The association patterns between serum biochemical markers and the numbers of stenotic coronary arteries were evaluated in a cross-sectional manner. Upon binary multiple logistic regression analysis, the risk factor patterns differed by gender. Age, high-density lipoprotein cholesterol (HDL) and homocysteine (HCY) were common risk factors for CHD in both males and females. Upon ordinal multiple logistic regression analysis, age, low-density lipoprotein cholesterol (LDL) and lipoprotein (Lp) (a) increased, and HDL decreased, as the number of stenotic coronary arteries increased in male patients. Age and Lp(a) were positively associated with the number of stenotic coronary arteries and total bilirubin (TBil) was negatively associated with the number of stenotic coronary arteries in female patients. Age and Lp(a) were common risk factors positively associated with the number of stenotic coronary arteries in both male and female patients. HDL and LDL were male-specific risk factors and TBil was a female-specific risk factor for an increasing number of stenotic coronary arteries. In conclusion, serum biomarker levels correlated with the number of stenotic coronary arteries and showed gender different patterns.

## INTRODUCTION

Coronary heart disease (CHD) refers to coronary artery stenosis and/or insufficient blood supply to the heart, which may lead to myocardial dysfunction and organic heart changes; CHD is thus also called ischemic cardiomyopathy [1–3]. CHD can result from a variety of coronary artery diseases, but the vast bulk of CHD is caused by coronary atherosclerosis [1–3]. The Global Burden of Disease report released by the World Health Organization in 2004 showed that ischemic cardiomyopathy was the leading cause of death, with 7.2 million deaths in 2004, accounting for as much 12.2% of all deaths worldwide [4].

Along with advances in our understanding of cardiology, CHD is now recognized as a complex and multifactorial disease [5]. Factors participating in the development of CHD include age, educational level, income, extent of physical activity, fat intake, overweight status, alcohol use, smoking, type II diabetes, cholesterol level, blood pressure, and the presence of other diseases such as obstructive sleep apnea [5–7]. These elements form a pathophysiological network in CHD; some elements of the network, such as high cholesterol, serve as relatively direct causes of CHD. On the other hand, some risks located further back in the causal network act indirectly through intermediary factors; these risks include physical inactivity, smoking, excessive fat intake, and obstructive sleep apnea [5].

Metabolic activity is essential for life, and metabolic disorders have been found to be associated with many diseases including CHD [8]. Many serum biochemical indices, which reflect dynamic physiological and pathophysiological processes within the body, have been found to be associated with CHD. The classic risk factors for CHD are cholesterol and triglyceride abnormalities, which constitute almost half of the population-attributable risk [9]. Patients with type II diabetes mellitus are considered to be at high risk of CHD regardless of other factors [10]. Increasing glycemic levels are positively correlated with vascular complications [11]. Recently, awareness of the importance of blood biochemical markers has grown and the number of risk factors for CHD has increased [12]. The estimated glomerular filtration rate derived from serum creatinine measurements has been found to be an important CHD risk factor in routine clinical practice [13]; also, homocysteine (HCY), uric acid (UA), microalbuminuria and cystatin C are now considered to be risk factors for CHD [14–17]. A significant correlation between serum GGT level and CHD has been reported [18]. Low bilirubin levels increased the coronary artery calcification risk in subjects with metabolic syndrome [19].

The above advances have greatly improved our knowledge on blood biochemical markers, however, they have not improved clinical practice in terms of CHD diagnosis or treatment as we expected. The correction of the guideline on the treatment of blood cholesterol to reduce atherosclerotic cardiovascular risk in adults is an example [20, 21]. Much work is required to enhance diagnostic and predictive strategies for CHD using blood biochemical markers. The work includes selection of the representative biochemical markers to avoid the confounding effect caused by the complex correlations between biochemical markers; clarification of the correlation patterns between blood biochemical markers and the lesion number of coronary arteries; stratification of gender as well as the evaluation on the associations between serum lipids and CHD under particular circumstances of extensive administration of lipid lowering drugs. In the present study, to evaluate the association between serum biomarkers and stenosis of the coronary arteries, 627 healthy controls and 1,049 CHD patients were sequentially recruited, representative biomarkers were selected by Pearson's correlation coefficients analysis, the associations between selected blood biochemical indices and the numbers of stenotic coronary arteries were evaluated by binary multiple logistic regression analysis as well as ordinal multiple logistic regression analysis.

## RESULTS

### Distribution of biochemical indices by the number of stenotic coronary arteries in males

As shown in Table 1, 400 male controls and 228, 204, 188 and 44 male patients with 1, 2, 3 and 4 stenotic coronary arteries, respectively, were recruited. The distribution tendencies of all variables except GGT differed between the case and control groups, and among the groups with 1, 2, 3 or 4 stenotic coronary arteries. The serum levels of UA, TC, TG, HDL-C, LDL-C, apoA, TBil, DBil and IBil were significantly lower in CHD patient groups than these in controls. Conversely, Age as well as the serum levels of apoB, Lp (a) and HCY were significantly higher in CHD patient groups than these in controls. Among CHD groups with 1, 2, 3 and 4 stenotic coronary arteries, the levels of all biochemical indices fluctuated between groups, while, some indices, such as Age LDL-C and Lp (a) showed an increasing tendency generally with the increase of stenotic coronary artery numbers.

**Table 1:** Demographic and clinical data (males)

Variable	Control	Patients (*n* = 664)				*P*
	(*n* = 400)	CAS1	CAS2	CAS3	CAS4	
		(*n* = 228)	(*n* = 204)	(*n* = 188)	(*n* = 44)	
Age (yr)	45.5 ± 12.1	60.3 ± 11.2	63.3 ± 11.2	62.7 ± 10.8	64.3 ± 9.1	<0.001
UA (umol/L)	404.9 ± 86.1	370.1 ± 96.8	387.8 ± 96.4	371.9 ± 92.0	370.6 ± 102.0	<0.001
TC (mmol/L)	4.9 ± 0.9	4.5 ± 1.1	4.7 ± 1.0	4.6 ± 1.1	4.4 ± 1.0	<0.001
TG (mmol/L)	1.9 (1.3, 3.1)	1.6 (1.2, 2.4)	1.6 (1.2, 2.5)	1.6 (1.1, 2.3)	1.6 (1.2, 2.1)	<0.001
HDL-C (mmol/L)	1.3 ± 0.3	1.1 ± 0.3	1.1 ± 0.3	1.1 ± 0.3	1.1 ± 0.2	<0.001
LDL-C (mmol/L)	3.0 ± 0.8	2.7 ± 1.0	2.9 ± 0.9	2.9 ± 1.0	2.8 ± 0.9	0.001
apoA (g/L)	1.4 ± 0.3	1.2 ± 0.2	1.2 ± 0.2	1.2 ± 0.2	1.2 ± 0.2	<0.001
apoB (g/L)	0.8 ± 0.2	0.9 ± 0.2	0.9 ± 0.2	0.9 ± 0.2	0.9 ± 0.2	<0.001
Lp(a) (mg/L)	110.0 (65.8, 208.3)	146.5 (61.0, 285.8)	164.0 (72.3, 304.5)	179.5 (91.8, 338.8)	248.5 (104.5, 403.8)	<0.001
HCY (umol/L)	11.9 (8.5, 14.9)	18.3 (14.9, 21.4)	18.4 (14.8, 22.0)	17.6 (13.7, 21.3)	20.9 (15.6, 27.8)	<0.001
TBil (umol/L)	13.6 (10.8, 17.6)	12.4 (9.2, 16.1)	12.0 (8.5, 15.9)	12.1 (9.5, 15.5)	11.2 (9.1, 14.8)	<0.001
DBil (umol/L)	3.5 (2.8, 4.5)	3.3 (2.4, 4.7)	2.9 (2.0, 4.3)	3.2 (2.1, 4.3)	2.3 (1.8, 3.8)	<0.001
IBil (umol/L)	10.0 (7.8, 12.9)	8.9 (5.9, 12.0)	8.7 (5.9, 12.2)	8.7 (6.4, 11.5)	8.4 (7.1, 12.0)	<0.001
GGT (IU/L)	36.0 (21.0, 63.8)	38.0 (26.0, 60.8)	36.0 (24.0, 58.8)	37.0 (24.0, 58.8)	36.5 (29.3, 57.8)	0.769

Normally distributed data are presented as means ± standard deviation (SDs) and skewed data as medians (with interquartile range). Differences among groups were compared using the Kruskal-Wallis H test or one-way ANOVA depending on the data distribution. Abbreviations: CAS1, 2, 3 and 4, patients with 1, 2, 3 or 4 stenotic coronary arteries; UA, uric acid; TC, total cholesterol; TG, triglyceride; HDL-C, high-density lipoprotein cholesterol; LDL-C, low- density lipoprotein cholesterol; apoA, apolipoprotein A; apoB, apolipoprotein B; Lp(a), Lipoprotein(a); HCY, homocysteine; TBil, total bilirubin; DBil, direct bilirubin; IBil, indirect bilirubin; GGT, gamma-glutamyl transpeptidase.

### Distribution of biochemical indices by the number of stenotic coronary arteries in females

As shown in Table 2, totals of 227 female controls and 170, 94, 105 and 16 female patients with 1, 2, 3 and 4 stenotic coronary arteries, respectively, were recruited. The distribution tendencies of all variables except LDL-C differed between the case and control groups, and among the groups with 1, 2, 3 or 4 stenotic coronary arteries. The serum levels of HDL-C, apoA, TBil and IBil were significantly lower in CHD patient groups than these in controls. Conversely, Age as well as the serum levels of UA, TC, TG, apoB, Lp (a), HCY, DBil and GGT were significantly higher in CHD patient groups than these in controls. Compare to males, the distribution tendencies of biochemical indices have changed considerably. These changes mainly manifested as: UA, TC, TG and DBil represented an opposing distribution trend; GGT level showed significant difference between the case and control groups only in females; and LDL-C level showed significant difference between the case and control groups only in males. Similar to these in males, although the levels of all biochemical indices fluctuated between CHD groups with 1, 2, 3 and 4 stenotic coronary arteries, some indices, such as age and Lp(a), tended to increase as the number of stenotic coronary arteries increased.

**Table 2:** Demographic and clinical data (females)

Variable	Control	Patients (*n* = 385)				*P*
	(*n* = 227)	CAS1	CAS2	CAS3	CAS4	
		(*n* = 170)	(*n* = 94)	(*n* = 105)	(*n* = 16)	
Age (yr)	43.3 ± 10.4	62.5 ± 9.0	65.8 ± 8.6	67.0 ± 8.5	70.9 ± 5.9	<0.001
UA (umol/L)	283.0 ± 60.0	323.9 ± 84.4	321.0 ± 85.6	325.2 ± 95.4	375.3 ± 79.4	<0.001
TC (mmol/L)	4.5 ± 0.8	4.9 ± 1.1	5.1 ± 1.3	5.0 ± 1.4	5.5 ± 1.4	<0.001
TG (mmol/L)	1.2 (0.9, 1.7)	1.9 (1.4, 2.7)	1.6 (1.2, 2.4)	1.7 (1.3, 2.2)	2.2 (1.9, 2.8)	<0.001
HDL-C (mmol/L)	1.6 ± 0.3	1.2 ± 0.3	1.2 ± 0.2	1.2 ± 0.3	1.2 ± 0.2	<0.001
LDL-C (mmol/L)	2.7 ± 0.7	2.8 ± 0.8	2.9 ± 0.9	2.8 ± 1.0	3.3 ± 1.5	0.073
apoA (g/L)	1.6 ± 0.3	1.3 ± 0.2	1.3 ± 0.3	1.2 ± 0.3	1.2 ± 0.2	<0.001
apoB (g/L)	0.8 ± 0.1	0.9 ± 0.2	0.9 ± 0.2	0.9 ± 0.3	1.0 ± 0.3	<0.001
Lp(a) (mg/L)	116.0 (70.0, 228.0)	122.0 (57.8, 334.8)	199.0 (95.5, 381.8)	190.0 (97.0, 408.5)	421.0 (168.8, 708.0)	<0.001
HCY (umol/L)	7.1 (4.3, 9.7)	16.0 (12.0, 19.4)	17.0 (12.4, 18.9)	16.0 (13.0, 20.3)	19.3 (16.5, 21.7)	<0.001
TBil (umol/L)	11.5 (8.9, 14.5)	11.3 (7.9, 14.0)	9.9 (7.5, 13.1)	9.1 (7.1, 12.4)	6.7 (5.6, 11.7)	<0.001
DBil (umol/L)	2.9 (2.3, 3.7)	3.7 (2.4, 4.7)	3.5 (2.5, 4.5)	3.2 (2.3, 4.1)	2.9 (2.0, 3.7)	0.011
IBil (umol/L)	8.5 (6.6, 10.8)	7.7 (5.2, 10.0)	6.1 (4.5, 9.1)	6.1 (3.5, 7.8)	4.9 (2.6, 8.3)	<0.001
GGT (IU/L)	15.0 (11.0, 22.0)	32.0 (20.0, 59.3)	30.5 (20.0, 49.8)	32.0 (20.5, 63.0)	44.5 (28.5, 75.5)	<0.001

Normally distributed data are presented as means ± standard deviation (SDs) and skewed data as medians (with interquartile range). Differences among groups were compared using the Kruskal-Wallis H test or one-way ANOVA depending on the nature of data distribution. For abbreviations, see Table 1.

### The correlation patterns among biochemical indexes

To explore correlations among biochemical indexes and to avoid the confounding effect caused by the complex correlation between biochemical markers for further analysis, the correlative patterns between biochemical indexes were quantified using Pearson correlation coefficients analysis. Serum lipid and bilirubin exhibited high Pearson correlation coefficients to indices belonging to the same biochemical classifications (Table 3). Of males, age, UA, TG, Lp(a), HCY and GGT were relatively independent of other variables; of females, UA, Lp(a) and GGT were also relatively independent of other variables. Age and TG showed relative high Pearson correlation coefficients to HCY (0.55) and HDL-C (0.41) in females, respectively (Table 3). Although Pearson correlation coefficient array displayed gender difference, the correlation patterns of indexes exhibited high Pearson correlation coefficients (> 0.40) were notably stable in both males and females. For example, TBil exhibited high Pearson correlation coefficients with DBil and IBil both in males and females (Table 3).

**Table 3:** Pearson's correlation coefficient analysis between the variables

*Males*														
	Age	UA	TC	TG	HDL-C	LDL-C	apoA	apoB	Lp(a)	HCY	TBil	DBil	IBil	GGT
Age	1.00													
UA	−0.16	1.00												
TC	−0.15	0.10	1.00											
TG	−0.21	0.21	0.31	1.00										
HDL-C	−0.09	−0.02	0.28	−0.26	1.00									
LDL-C	−0.07	0.03	**0.69**	−0.14	0.16	1.00								
apoA	−0.18	0.07	0.31	0.05	**0.80**	0.04	1.00							
apoB	−0.27	0.12	**0.74**	0.23	0.11	**0.66**	0.23	1.00						
Lp(a)	−0.05	0.04	0.16	−0.02	0.16	0.13	0.15	0.19	1.00					
HCY	0.34	0.07	−0.07	−0.10	−0.17	−0.01	−0.24	−0.11	−0.06	1.00				
TBil	−0.10	−0.03	−0.02	−0.12	0.15	0.01	0.13	0.03	0.00	−0.07	1.00			
DBil	0.02	−0.05	−0.17	−0.24	0.11	−0.13	0.01	−0.18	−0.04	−0.01	**0.70**	1.00		
IBil	−0.13	−0.02	0.04	−0.06	0.14	0.07	0.15	0.11	0.02	−0.08	**0.96**	**0.47**	1.00	
GGT	−0.16	0.10	0.15	0.26	0.03	−0.01	0.13	0.12	−0.03	−0.02	0.03	0.04	0.03	1.00
*Females*														
	Age	UA	TC	TG	HDL-C	LDL-C	apoA	apoB	Lp(a)	HCY	TBil	DBil	IBil	GGT
Age	1.00													
UA	0.23	1.00												
TC	0.21	0.10	1.00											
TG	0.20	0.20	0.34	1.00										
HDL-C	−0.37	−0.19	0.12	−0.41	1.00									
LDL-C	0.16	0.06	**0.76**	−0.02	0.10	1.00								
apoA	−0.36	−0.17	0.14	−0.13	**0.81**	0.08	1.00							
apoB	0.12	0.10	**0.73**	0.32	−0.06	**0.73**	0.06	1.00						
Lp(a)	−0.05	−0.10	0.13	−0.08	0.13	0.16	0.07	0.17	1.00					
HCY	**0.55**	0.33	0.13	0.24	−0.29	0.02	−0.34	−0.01	−0.05	1.00				
TBil	−0.10	−0.08	0.01	−0.03	0.13	0.01	0.10	0.04	0.05	−0.10	1.00			
DBil	0.11	−0.01	−0.09	−0.14	−0.04	−0.13	−0.18	−0.18	−0.05	0.11	**0.68**	1.00		
IBil	−0.17	−0.09	0.05	0.02	0.18	0.07	0.21	0.13	0.08	−0.17	**0.95**	**0.41**	1.00	
GGT	0.27	0.15	0.10	0.15	−0.23	0.08	−0.25	0.08	−0.03	0.20	−0.05	0.10	−0.11	1.00

For abbreviations, see Table 1. Pearson's correlation coefficients were marked as bold when over 0.40.

The above analysis showed complex correlations among biochemical indexes and suggested the necessity of variable selection in further regression analysis. The principles of our variable selection for following analyses are as follows: because the relative independence, UA, Lp(a) and GGT were selected; age, as a classic unmodifiable risk factor [20], was also selected; HDL-C and LDL-C were selected to represent serum lipid profile; TBil was selected to represent bilirubin; HCY, was relatively independent of other variables in males, and hence was also selected. The correlation between HCY and age in females will be further observed in following logistic regression analysis.

### Factors associated with the presence of CHD

To define variables associated with the presence of CHD, and to determine if any between-gender differences existed, multivariable logistic regression analysis was first performed in a binary manner. Guided by the Pearson correlation coefficient analysis described above, recognized biochemical principles, and the fact that we were performing primary univariate analysis; the following indices: (age, UA, HDL-C, LDL-C, Lp(a), HCY, and TBil) and (age, UA, HDL-C, LDL-C, Lp(a), HCY, TBil and GGT), were selected as independent variables in binary multiple logistic regression analysis in males and females respectively. As showed in Table 4, in males, age, HCY and Lp (a) were positively associated with the presence of CHD in our multivariable logistic regression model; UA and HDL-C were negatively associated with the presence of CHD; in females, age, GGT and HCY were positively associated with the presence of CHD in our multivariable logistic regression model; HDL-C was negatively associated with presence of CHD. The risk factor patterns differed between males and females, but age, HDL-C and HCY were common risk factors for CHD both in males and females.

**Table 4:** Factors associated with the presence of CHD

Variable	OR	95% CI	*P*
*Males*			
Age	1.109	1.090 - 1.128	<0.001
HDL-C	0.042	0.021 - 0.084	<0.001
Lp(a)	1.002	1.001 - 1.003	<0.001
UA	0.996	0.994 - 0.999	<0.001
HCY	1.222	1.173 - 1.274	<0.001
*Females*			
Age	1.176	1.129 - 1.225	<0.001
HDL-C	0.037	0.011 - 0.125	<0.001
HCY	1.313	1.213 - 1.422	<0.001
GGT	1.017	1.004 - 1.030	0.009

OR, odds ratio; 95% CI, 95% confidence interval; for more abbreviations, see Table 1. ORs for continuous variables = the OR for an increase of 1 unit.

### Factors associated with the number of stenotic coronary arteries in CHD patients

Although serum biochemical indices associated with the presence of CHD have been frequently reported [5–19], factors associated with the number of stenotic coronary arteries in CHD patients are few in number. Our above analysis on the distributions of biochemical indices showed that the levels of some biochemical indices increased as the number of stenotic coronary arteries increased (Table 1 and 2). On the other hand, the levels of other biochemical indices decreased as the number of stenotic coronary arteries increased (Table 1 and 2). To explore whether there is a dose effect relationship existing between biochemical indices and the number of stenotic coronary arteries, multivariable logistic regression was further performed, in an ordinal manner, on data from the CHD patients.

As shown in Table 5, in male patients, age and the LDL-C and Lp(a) levels were positively associated with the number of stenotic coronary arteries increase with OR and 95% CI of 1.017 (1.003 - 1.030), 1.164 (1.035 - 1.358) and 1.001 (1.000 - 1.002), respectively; the HDL-C level was negatively associated with the number of stenotic coronary arteries increase with OR and 95% CI of 0.573 (0.301 - 0.937). In female patients, age and the Lp(a) level were positively associated with the number of stenotic coronary arteries increase with OR and 95% CI of 1.065 (1.040 - 1.090) and 1.002 (1.001 - 1.002), respectively; the TBil level was negatively associated with the number of stenotic coronary arteries increase with OR and 95% CI of 0.915 (0.876 - 0.955) (Table 5). Age and Lp (a) were common risk factors positively associated with an increase in the number of stenotic coronary arteries in both male and female CHD patients. Interestingly, the HDL-C and LDL-C, and the TBil, were male- and female-specific risk factors, respectively (Table 5).

**Table 5:** Factors associated with number of stenosis coronary arteries in CHD patients

Variable	OR	95% CI	*P*
*Male*			
Age	1.017	1.003 - 1.030	0.008
HDL-C	0.573	0.301 - 0.937	0.049
LDL-C	1.164	1.035 - 1.358	0.043
Lp(a)	1.001	1.000 - 1.002	0.002
*Female*			
Age	1.065	1.040 - 1.090	<0.001
Lp(a)	1.002	1.001 - 1.002	<0.001
TBil	0.915	0.876 - 0.955	<0.001

OR, odds ratio; 95% CI, 95% confidence interval; for more abbreviations, see Table 1. ORs for continuous variables = OR for an increase of 1 unit.

## DISCUSSION

In this study, we explored two important patterns of association between serum biomarker levels and CHD. First, we evaluated biochemical markers associated with the presence of CHD; such analysis is similar to that performed in most earlier relevant studies [5, 14, 22]. We clarified that the pattern of risk factors differed between males and females. Thus, age, UA, HDL-C, HCY and Lp(a) were associated with the presence of CHD in males; and age, HDL-C, GGT and HCY were associated with the presence of CHD in females. Age, HDL-C and HCY were common risk factors for CHD in both males and females. Although our findings on the importance of age, HDL-C and HCY are consistent with those of other reports [5, 14, 22], our data on UA and GGT differ from these reports. In terms of UA, any association with CHD remains controversial. Some reports found that UA was a dependent risk factor for CHD [23]; Tuttle et al. showed that the serum UA level was linearly related to CAD severity in females [24], but a recent meta-analysis found that the UA level was unlikely to usefully enhance the prediction of CHD [25]. Our results show that UA was negatively associated with the presence of CHD in males; the odds ratio (OR) was 0.996. The status of GGT as a risk factor for CHD is also unclear [26, 27]. Our results show that GGT is an independent risk factor for CHD in females in the binary analysis. The contradictory results might be caused by differences in race, sample size, and/or the extent of control of confounding factors. As we fully considered multicollinearity when calculating sample sizes, we believe that our results are closer to the real associations between serum biomarker levels and the presence of CHD.

Secondly, we evaluated biochemical markers associated with the number of stenotic coronary arteries. We used ordinal multivariable logistic regression modeling to this end; such analysis, is always used to reveal a dose effect relationship between independent variables and dependent variables, has rarely been adopted previously. We showed that patient age, LDL-C and Lp(a) increased, and HDL-C decreased, as the number of stenotic coronary arteries increased in male patients. Both age and Lp(a) were positively associated with the number of stenotic coronary arteries whereas TBil was negatively associated with the number of stenotic coronary arteries in female patients. Although Lp(a) was not an independent risk factor for CHD in females upon binary multivariable logistic regression analysis, Lp(a) and age were risk factors positively associated with stenotic coronary artery numbers in both male and female patients. When we reviewed the distribution of Lp(a) (Table 1 and 2), we noted that Lp(a) gradually increased from patients with one stenotic coronary artery to patients with four stenotic coronary arteries both in male and female patients. This aspect of Lp(a) distribution explained why Lp(a) was an independent risk factor for stenotic coronary artery numbers both in female and male patients upon ordinal analysis. As for why Lp(a) was not an independent risk factor for females with CHD in binary logistic regression analysis, the correlation coefficients of Lp(a) with HDL-C and LDL-C were higher in males than that in females (Table 3) and which might affect the selection of ariables in the final equation in binary logistic regression analysis. As the levels of biochemical markers associated with the number of stenotic coronary arteries were evaluated using ordinal multivariable logistic regression modeling (which has seldom been used in earlier work), the associations between serum biomarker levels and the number of stenotic coronary arteries revealed in this report advance our knowledge on the role played by metabolic disorders in the pathogenesis of CHD.

Recent studies have suggested that a high level of bilirubin is associated with favorable coronary endothelial function, and that a strong, inverse independent relationship exists between conjugated bilirubin level and coronary artery calcium score [27, 28]. These reports support a protective role for bilirubin in the pathogenesis of CHD. Although Sung et al. recruited 14,583 subjects, the proportion of subjects with elevated coronary artery calcium levels was not sufficiently large (totals: males = 1,351, females = 111). Furthermore, the cited authors did not evaluate association patterns by gender [29], and thus did not derive distinct ORs linking bilirubin levels with CHD in males and females. We have shown here that TBil is a female-specific factor negatively associated with an increase in the number of stenotic coronary arteries.

Of the lipids profile, existing knowledge believe that, TC and LDL-C levels are positively associated with the presence of CHD, HDL-C is negatively associated with the occurrence of CHD and the role of TG in the association with CHD is in dispute [30–32]. There are some differences between our data and consensus. TC and TG represented an opposing distribution trend in our population, of males, TC and TG levels were higher in control than in CHD patients, however, this phenomenon is just the opposite of that in the female population. In addition, the LDL-C level was lower in male CHD patients than that in male control. We think that the commonly used lipid lowering therapy may be one of the reasons to explain this “abnormal phenomenon”. Although we were hard to exclude subjects with lipid lowering therapy at the beginning of this study, our ordinal multivariable logistic regression analysis performed on data from the CHD patients showed that, of male patients, LDL-C and Lp(a) were positively associated with, and HDL-C was negatively associated with, an increase in the number of stenotic coronary arteries; of female patients, Lp(a) was positively associated with an increase in the number of stenotic coronary arteries. These data suggested that lipids still played important role in the development of CHD regardless of lipid lowering drugs are widely used.

The limitations of our report are, first, that the control populations were significantly younger than the CHD patients; this may influence the associations seen between serum biomarkers and the presence of CHD. However, upon ordinal multivariable logistic regression modeling, only CHD patients were included, so the age difference between controls and patients was irrelevant. Second, many other risk factors, such as serum glucose level, body mass index, and smoking and drinking status, were not evaluated, because the work was retrospective in nature. Third, more than 90% patient were treated with lipid lowering drugs, we could not exclude these patients. Although our data might not reflect the associations between serum lipids and CHD in patients without lipid lowing therapy, our report evaluated the associations between serum biomarkers and CHD in patients with lipid lowing therapy and which is the significance of our study.

In conclusion, age and Lp(a) increased with the number of stenotic coronary arteries in both male and female patients; HDL-C and LDL-C were male-specific risk factors for an increasing number of stenotic coronary arteries; TBil was a female-specific risk factor for an increasing number of stenotic coronary arteries.

## MATERIALS AND METHODS

### Ethical issues

This study was conducted according to the World Medical Association (WMA) Declaration of Helsinki - Ethical Principles for Medical Research Involving Human Subjects (http://www.wma.net/en/30publications/10policies/b3/). The protocol was approved by the Review Board of the People's Hospital of Yuxi City (Yuxi, China, approval number: YNYXH2010-0012). All subjects provided written informed consent prior to participating in this study. The participants were simultaneously informed of their right to withdraw consent given by themselves or their kin, caretakers, or guardians.

### Subjects

As we reported previously [33], unrelated sequential patients visiting the People's Hospital of Yuxi City were recruited between September 2010 and July 2013. The diagnosis of CHD was based on the 2012 ACCF/AHA/ACP/AATS/PCNA/SCAI/STS guideline for the diagnosis and management of patients with stable ischemic heart disease [34]. Patients suspected of having CHD were initially screened based on their medical and family histories, risk factors for CHD, and physical examination. All CHD patients were confirmed to have stenosis (narrowing of the luminal diameter > 50%) within one of the left main coronary arteries, the anterior descending branch, and/or the right coronary artery, caused by atheromatous plaque, as revealed by coronary angiography. Patients who met the following exclusion criteria were excluded from the study: alcohol abuse; a history of diabetes; a history of smoking; evidence of non-coronary atherosclerotic disease; and/or presence of a chronic infectious disease, chronic or acute liver disease, chronic lung disease, chronic kidney disease, chronic wasting disease, a malignant tumor, an autoimmune disease, or xanthelasma. It should be noted that we could not exclude the patients with lipid lowering therapy due to the extensive administration of lipid lowering drugs. At the same time, individuals with no history of CHD and no latent CHD, as revealed by two or more cardiologic tests, were recruited as controls.

### Laboratory-based biochemical testing

As we reported previously [33], a fasting blood sample was collected from each participant *via* the antecubital vein in the morning of the day on which he or she visited our hospital. Serum lipid profiles, including triglycerides (TG), total cholesterol (TC), high-density lipoprotein-cholesterol (HDL-C), low-density lipoprotein-cholesterol (LDL-C), and apolipoprotein (apo)A, apoB, apoE, and lipoprotein (Lp) (a); indicators of kidney function, including UA; indicators of liver function, including gamma-glutamyl transpeptidase (GGT), total bilirubin (TBil), indirect bilirubin (IBil), and direct bilirubin (DBil); and HCY levels, were measured in our hospital's laboratory using routine procedures.

### The questionnaire

A questionnaire was self-completed by all participants; the items explored included ethnicity, age, sex, obesity, family history, medical history, manifestations of CHD, and behavioral risk factors such as smoking status, alcohol abuse and high dietary intakes of high-fat, high-carbohydrate and high-calorie diets.

### Statistical analysis

All analyses were performed using SPSS for Windows software (ver. 18.0; SPSS Inc., Chicago, IL, USA). Continuous variables that were normally (or approximately normally) distributed are presented as means ± standard deviation (SD). Variables with skewed distributions are presented as medians (with the 1st and 3rd quantiles). Differences in demographic and clinical data among groups were explored using the Kruskal-Wallis H test or one-way ANOVA, depending on the distribution of the data. To eliminate multicollinearity between or among variables, Pearson's correlation coefficients were calculated prior to further statistical analysis. To define the risk factors associated with the presence of CHD, multiple unconditional logistic regression modeling served as the primary analysis of the independent effects of each variable. Potentially relevant variables identified in the univariable analysis (*P* < 0.10) were introduced into the initial model and then eliminated manually, using the backward step-by-step approach, with reference to the largest *P*-value. All factors showing statistical significance at *P* < 0.05 were retained in the final predictive model. To evaluate associations between risk factors and the number of stenotic coronary arteries, a multiple unconditional logistic regression model was used to evaluate data from CHD patients in an ordinal manner. The regression coefficients, and the 95% confidence intervals (CIs) of risk factors that significantly influenced the number of stenotic coronary arteries, were calculated. The significance level (alpha) was set at 0.05.
